# Function of small GTPases in *Dictyostelium* macropinocytosis

**DOI:** 10.1098/rstb.2018.0150

**Published:** 2018-12-17

**Authors:** Thomas D. Williams, Peggy I. Paschke, Robert R. Kay

**Affiliations:** MRC-Laboratory of Molecular Biology, Francis Crick Avenue, Cambridge CB2 0QH, UK

**Keywords:** Ras, Rac, small G-protein, macropinocytosis, endocytosis, *Dictyostelium discoideum*

## Abstract

Macropinocytosis—the large-scale, non-specific uptake of fluid by cells—is used by *Dictyostelium discoideum* amoebae to obtain nutrients. These cells form circular ruffles around regions of membrane defined by a patch of phosphatidylinositol (3,4,5)-trisphosphate (PIP3) and the activated forms of the small G-proteins Ras and Rac. When this ruffle closes, a vesicle of the medium is delivered to the cell interior for further processing. It is accepted that PIP3 is required for efficient macropinocytosis. Here, we assess the roles of Ras and Rac in *Dictyostelium* macropinocytosis. Gain-of-function experiments show that macropinocytosis is stimulated by persistent Ras activation and genetic analysis suggests that RasG and RasS are the key Ras proteins involved. Among the activating guanine exchange factors (GEFs), GefF is implicated in macropinocytosis by an insertional mutant. The individual roles of Rho family proteins are little understood but activation of at least some may be independent of PIP3.

This article is part of the Theo Murphy meeting issue ‘Macropinocytosis’.

## 1. Introduction

Macropinocytosis is an actin-driven process in which cup-shaped projections several microns across are extended from the plasma membrane, then close to form macropinosomes and deliver a vesicle filled with extracellular fluid to the cell interior [1,2]. It has been described in a variety of metazoa, including mammals, flies and nematode worms as well as in amoebae such as *Dictyostelium discoideum* and the pathogenic *Entamoeba histolytica*. The evolutionary origin of macropinocytosis lies at least as far back as the common ancestor of the Metazoa and Amoebozoa [3]. Macropinocytosis is of considerable medical importance having been implicated in feeding by some cancer cells, host invasion by pathogens, antigen sampling by immune cells and the progression of neurodegenerative disease [4].

*Dictyostelium* is a soil-living amoeba whose life cycle alternates between solitary growth on bacteria or, when starved, collective development into stalked fruiting bodies, and more rarely into sexual macrocysts [5,6]. It is an avid phagocyte and prefers to consume bacteria when these are available, but in their absence can also use macropinocytosis to feed on liquid medium [7,8]. Laboratory strains have a special propensity for macropinocytosis (see later), which accounts for the great majority of their fluid uptake and is readily measured using fluorescent dextran and flow cytometry [7–9]. Macropinocytosis is constitutive in liquid culture and does not need to be stimulated by growth factors. *Dictyostelium* cells are also easy to observe and manipulate and amenable to genetic screens making them a valuable model for investigating the core mechanisms of macropinocytosis.

## 2. Macropinocytic cups and patches

To form and close a macropinocytic cup, actin dynamics must be regulated over a scale of several microns and times of a few minutes. A ring of protrusive actin is created under the plasma membrane to extend the walls of the macropinocytic cup and, at some point, the cup must stop extending and close to form a vesicle, which then rapidly loses its F-actin coat. We have suggested that the ‘macropinocytic patch’ of signalling molecules lying within the cup produces this spatial organization through recruiting effector molecules that control where and when actin polymerizes and depolymerizes [10].

**Figure 1.**
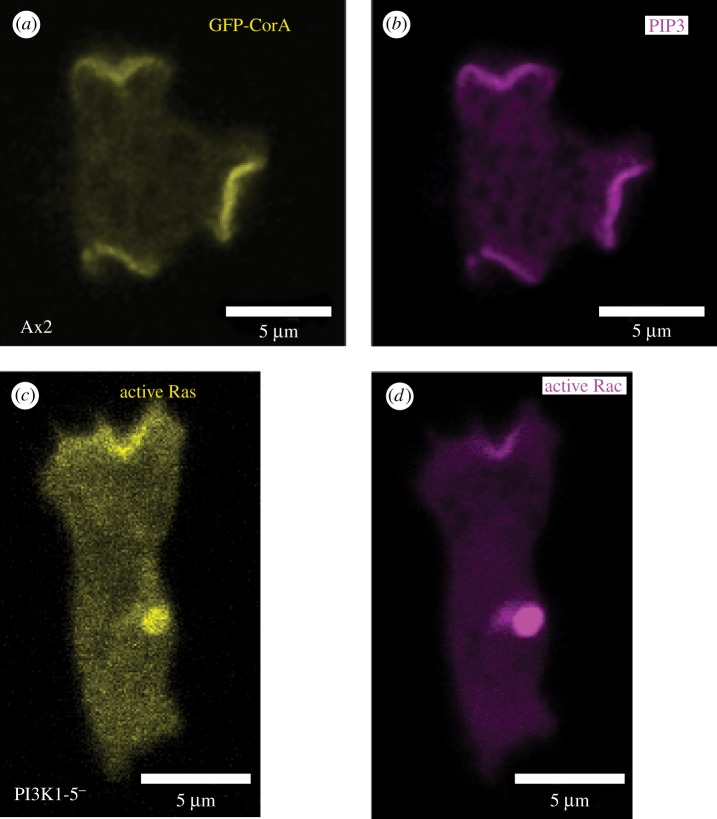
Macropinocytic signalling patches in the plasma membrane of *Dictyostelium* cells. (*a,b*) The F-actin-binding protein coronin (yellow) [11] and PIP3 reporter (magenta) co-localize at macropinocytic patches in vegetative Ax2 cells expressing a dual reporter plasmid (pPI32) for GFP-CorA and PH-Akt-mCherry (PIP3-binding). (*c,d*) Active Ras (yellow) and Rac (magenta) patches still form in a mutant lacking all Ras-activated PI3-kinases and having only 10% of normal PIP3 levels [12,13]. PI3K1-5− cells expressing a dual reporter plasmid pDM1140 for Raf1-RBD-mCherry (binds Ras-GTP) and PakB-CRIB-GFP (binds Rac-GTP) were grown on bacteria and adapted to HL5 liquid medium over night for imaging.

Macropinocytic patches were discovered in growing *Dictyostelium* cells using pleckstrin homology (PH)-domain reporters for phosphatidylinositol (3,4,5)-trisphosphate (PIP3) [14,15]. They are discrete areas in the plasma membrane of strong reporter recruitment, microns across, with relatively sharp boundaries that are associated with F-actin projections and are shown in this case by reporters for active-Ras and the actin-binding protein coronin (figure 1*a,b*) [16]. These projections extend and seal forming internal vesicles, confirming their macropinocytic nature. Similar patches in starving cells have been linked to chemotaxis [14,17–19], but they all share a common organization and we consider them as macropinocytic structures [10]. Mammalian cells also produce patches of PIP3 in macropinocytic cups, which become prominent when linear ruffles circularise [20,21].

PIP3 patches coincide with patches of activated Ras and Rac forming a spatially restricted signalling network [10,22,23], from which the lipid phosphatase PTEN, which converts PIP3 back to PI(4,5)P2, is excluded [12,24]. PIP3 patches are sensitive to inhibition of actin dynamics with latrunculin-A [22], and we find that they are sensitive to other cytoskeletal inhibitors as well, suggesting that cytoskeletal function is essential for their formation (Peggy I. Paschke, unpublished observations).

Macropinocytic cups are formed from sheets and spikes of F-actin [7,10,25,26] suggesting that both the Arp2/3 complex and formins initiate actin polymerization, and indeed both are recruited to cups [27,28]. PIP3 patches recruit SCAR/WAVE and WASP—activators of the Arp2/3 complex—to their periphery, but not their centre [10]. These activators are expected to trigger actin polymerization in a ring, thus shaping the walls of the macropinocytic cup.

The microscopic examination of macropinocytic cups has to an extent outrun functional analysis of their components. PIP3 is required for efficient macropinocytosis in both mammalian and *Dictyostelium* cells as shown by inhibitor and gene knock-out experiments [12,29,30]. Though *Dictyostelium* phosphoinositides are unusual in having an ether-linked lipid tail [31], this makes no known difference to the functions of the phospho-inositide head group. *Dictyostelium* has five PI3-kinases with Ras-binding domains (RBDs), similar to class-1 PI3-kinases in mammalian cells, but differing in that regulatory subunits have not been reported [13,32]. Mutation of the RBD and Ras-binding experiments suggests that these enzymes are directly activated by Ras [33]. A double knock-out mutant shows that PI3K1 and PI3K2 produce most of the PIP3 in growing cells and are required to form PIP3 patches and for most of the cellular fluid uptake [12,31]. Knock-out of PI3K4 alone also impairs fluid uptake without much affecting PIP3 levels, suggesting that it has a separate and specialist function. Knock-out of the PTEN phosphatase results in cells with very high levels of PIP3 and greatly inhibits macropinocytosis [10]. Once a macropinosome is internalized, PI(3,4,5)P3 is rapidly converted to PI(3,4)P2 and then, very likely, to PI(3)P [15]. This progression of PIP species is necessary for efficient macropinocytosis [34].

With the importance of PIP3 in macropinocytosis well established, here we focus on the small G-proteins of the macropinocytic patch—proteins of the Ras and Rho families—that likely act as activators and effectors for PIP3.

## 3. Material and methods

*Dictyostelium discoideum* strain Ax2 (Kay laboratory) was grown in HL5 liquid medium (Formedium) or on *Klebsiella pneumonia* bacteria on SM agar plates (Formedium) at 22°C and regularly renewed from frozen stocks. Strains are described in electronic supplementary material, table S1. DdB is a non-axenic strain derived from NC4 in the Sussman laboratory and the parent of the standard axenic strains [35]. Where necessary, cells were freed of growth medium or bacteria by low speed centrifugation (300 *g* × 3 min) and washing in KK2 (16.6 mM KH_2_PO_4_, 3.8 mM K_2_HPO_4_, 1 mM MgSO_4_, pH 6.1).

Since many macropinocytic mutants grow poorly in liquid medium and may accumulate suppressor mutations, molecular genetic manipulations were usually performed using selection on bacteria as food source, with a new family of vectors (many available from Addgene) and square-wave electroporation (BioRad Xcell Gene Pulser, 0.2 mm gap cuvette, 2 pulses of 350 V and 8 ms separated by 1 s) [36,37]. Insertion and deletion mutants were identified by diagnostic PCR from genomic DNA using primers both within and without the homology region of the plasmid. Reporter construct coding sequences were verified by DNA sequencing. Where necessary, the selectable marker was removed using the Cre/Lox system [38]. Plasmid and primer details are in electronic supplementary materials, tables S2 and S3, with example strain verification in electronic supplementary materials, figure S1.

Macropinocytosis was measured as total fluid uptake by flow cytometry [8,39]. Generally, cells were grown on bacteria, then adapted for about 24 h in 96-well plates in HL5 without bacteria to upregulate macropinocytosis; further upregulation is sometimes obtained by supplementing the HL5 with 10% fetal calf serum (FCS), which was used as indicated. Adapted cells were incubated with 0.5 mg ml^−1^ TRITC dextran (155 kDa; Sigma) for 1 h, washed, stopped and detached with azide, and their fluorescence analysed by flow cytometry. Error bars are the standard error throughout; statistical significance was estimated by unpaired two-tailed *t*-tests: *p < 0.1, **p < 0.05, ***p < 0.01.

Where a low fluorescent background was required, microscopy was performed with cells incubated for at least 1 h in either LowFlo (Formedium) or Simple Up Regulation medium (SUM: KK2, 0.1 mM CaCl_2_, 55 mM glucose, 4 mM arginine, 3.7 mM glutamate, 8.5 mM lysine, pH 6.5 [8]) as indicated (these both maintain the rate of macropinocytosis for prolonged periods). The rate of macropinosome formation was measured by upregulating cells in HL5, then switching to SUM for about an hour before pulsing for 1 min with FITC-dextran, fixing with 4% paraformaldehyde and counting the number of macropinosomes per cell by microscopy [8]. The rate of macropinocytic patch formation was measured in single confocal sections of live cells using a PH-PkgE-mCherry marker for PIP3, over a period of 3–5 min [8]. Cells were observed using a Zeiss 700 series confocal microscope with the indicated reporters.

## 4. The role of Ras proteins in macropinocytosis

Small G-proteins, including Ras and Rac, are molecular switches that can bind either GDP (off) or GTP (on). They are turned on by guanine exchange factors (GEFs, swap GDP for GTP) and off by GTPase activating proteins (GAPs, promote the hydrolysis of GTP to GDP). Mutation of RasGEFs should decrease Ras activity and of RasGAPs, increase it. Ras can also be activated directly by mutation, as in many cancers, where by far the most frequent activating mutation is the G12T substitution.

Ras was first implicated in macropinocytosis in mammalian cells more than 30 years ago. Ras can be activated by growth factors, which also stimulate macropinocytosis and landmark experiments showed that injecting activated (oncogenic) Ras into fibroblasts stimulates ruffling and macropinocytosis [40]. However, an essential role for Ras has been challenged by recent work showing that murine embryonic fibroblasts in which K-Ras, H-Ras and N-Ras are mutated can still carry out macropinocytosis [41].

## 5. Genetic activation of Ras stimulates macropinocytosis in *Dictyostelium*

Ras has also been studied in *Dictyostelium* for more than 30 years [42], but rarely in the context of macropinocytosis. *Dictyostelium* has an expanded Ras family of 14 proteins encoded in the genome, with RasG being the most highly expressed in growing cells [43,44] and having 71% identity to human K-Ras. There is also a highly conserved and expressed homologue of Rap [45]. Ras effectors include the TORC2 complex and five PI3-kinases with RBDs [12,33]. Ras is activated by the chemoattractants cyclic-AMP and folic acid acting through their G-protein coupled receptors, and although this stimulates PIP3 production, macropinocytosis is not stimulated (Thomas D Williams, unpublished observations). However, a link is shown by two gain-of-function experiments, in which persistent activation of Ras stimulates macropinocytosis.

In the first experiment, Ras is activated by deleting a RasGAP. In the late 1960s, *Dictyostelium* mutants were selected that could grow in liquid culture without the presence of bacteria (axenically) [46,47]. These strains thrived due to their vigorous macropinocytic uptake of the medium. The causative mutation was eventually tracked down to deletion of the RasGAP, NF1 [48]. Inactivating NF1 results in cells forming larger and more frequent Ras/PIP3 patches in the plasma membrane and an increase in fluid uptake of up to 20-fold.

**Figure 2.**
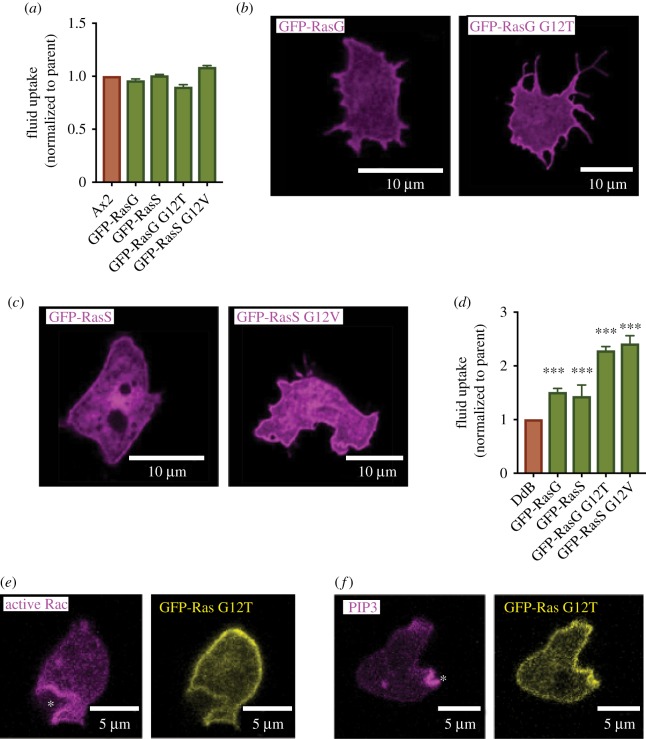
Macropinocytosis is stimulated by expressing activated-Ras in wild-type cells. (*a*) Expression of RasG or RasS or their activated versions (as GFP fusions) fails to stimulate macropinocytosis in Ax2 cells, where Ras is already somewhat activated due to deletion of the RasGAP NF1. (*b*,*c*) The GFP-Ras proteins are targeted to the plasma membrane of wild-type DdB cells as expected. (*d*) Expression of Ras and especially activated-Ras stimulates macropinocytosis in wild-type DdB cells, which have intact NF1. (*e*,*f*) Though the activated GFP-Ras proteins are recruited fairly uniformly to the plasma membrane of Ax2 cells, PIP3 and active-Rac accumulate locally at macropinocytic cups (asterisks). Ras was expressed using pPI432 (GFP-RasG), pPI332 (GFP-RasG G12T), pPI434 (GFP-RasS) or pPI445 (GFP-RasS G12V). Active Rac was visualised using PakB-CRIB-mCherry (pTW050) and PIP3 using PH-PkgE-mCherry (pPI363). DdB cells grown on bacteria were adapted in HL5+10% FCS over-night before fluid uptake assay or imaging.

In the second experiment, which we report here, an activated version of Ras is expressed. In early experiments, activated Ras expression caused complex phenotypic effects in axenic strains of *Dictyostelium* but did not stimulate growth [49–51] and we can confirm that macropinocytosis is not stimulated either (figure 2*a*).

In hindsight, this experiment might have failed due to the use of axenic cells in which NF1 is deleted and Ras partially activated. Macropinocytosis might already be maximal in these cells, or further activation of Ras might be deleterious. We therefore repeated the experiment in wild-type DdB cells with intact NF1, using transformation methods for non-axenic cells [36]. In this case, the outcome is different: expressing wild-type Ras (RasG or RasS, which have been linked to macropinocytosis; see later) in DdB cells already causes a modest increase in fluid uptake, while expressing the activated forms causes fluid uptake to more than double (figure 2*d*). Ras proteins are correctly targeted to the plasma membrane in all cases and even though they appear fairly evenly distributed, discrete patches of active Rac and PIP3 form and close as macropinosomes (figure 2*b*,*c*,*e*,*f*).

These gain of function experiments show that active Ras can stimulate macropinocytosis in *Dictyostelium* amoebae as it does in mammalian cells, but do not show which are the relevant Ras proteins.

## 6. The role of individual Ras proteins in macropinocytosis

**Figure 3.**
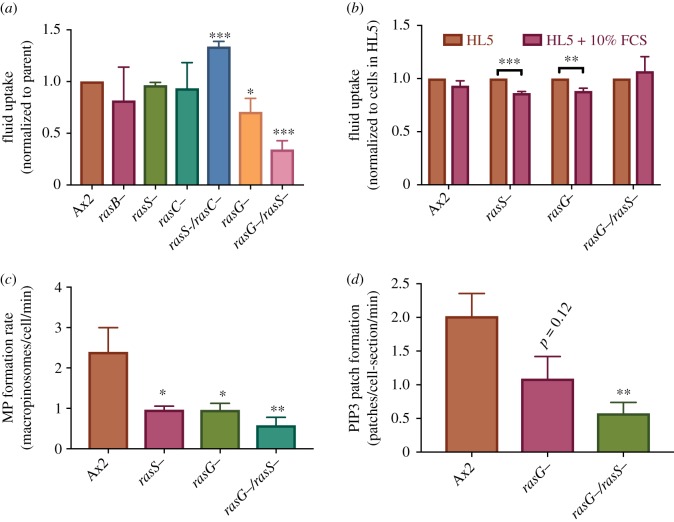
Identification of Ras proteins important in macropinocytosis. (*a*) Fluid uptake by Ras mutants: fluid uptake is significantly reduced in a RasG− mutant and strongly in a RasG−/RasS− double mutant. (*b*) Fluid uptake by selected Ras mutants adapted either in HL5 or HL5 + 10% FCS. The reinforced HL5 + 10% medium gives similar results to HL5 (results for each strain are normalized to the same strain in HL5). (*c*) The rate of macropinosome formation is greatly decreased in RasG− and RasS− mutants. (*d*) The rate of macropinocytic patch formation is reduced in RasG− and RasG−/S− mutants.

Only four Ras proteins (RasG, S, B and C) along with the closely related Rap protein, RapA, are substantially expressed in growing cells [43,44]. RasD expression strongly increases during development, and it is required for phototaxis of migrating slugs (motile aggregates of up to 10^5^ cells) [52]. Although RasC is expressed in growing cells, its clearest role is during the aggregation stage of development, when it is required to produce relayed cyclic-AMP signals [53]. RasC null mutants have either modestly increased or normal fluid uptake [54] (figure 3*a*). Mutants have been made in RasX, RasY and RasZ with no obvious phenotypes noted [55]. These minor and somewhat divergent Ras proteins may be important in other phases of the life cycle, such as sexual development, but seem irrelevant for macropinocytosis, except for the possibility of compensatory upregulation, as occurs with RasD when RasG is deleted [56].

PI3-kinases 1, 2 and 4 are the most important for macropinocytosis and can be activated by Ras through their RBDs [33]. Binding studies show that the activated (GTP-bound) forms of RasG and RasS bind the RBD domains of these three macropinocytic PI3-kinases well, whereas RasB and RapA prefer PI3K3 and PI3K4 [12,57]. RasC does not appear to bind any of the PI3-kinases but does bind the TORC2 complex, along with RapA [58].

Ras mutants have been isolated in different genetic backgrounds [35], using axenic growth conditions that favour the selection of suppressors of macropinocytic defects. Not surprisingly, there are inconsistencies in the macropinocytic phenotypes. To address this issue, we have remade some key mutants in a uniform genetic background using a transformation method not requiring axenic growth [36], then measured their fluid uptake using fluorescent dextran and flow cytometry (figure 3*a*,*b*) [8]. For these comparisons, cells were grown on bacteria and transferred to the axenic medium to upregulate macropinocytosis (by 10–20 fold in Ax2 cells). As upregulation depends on the nutritional content of the medium and some strains upregulate even better in medium enriched with 10% FCS, this also was tested [8].

RasG is the most highly expressed Ras protein [44] and seems the most important for macropinocytosis. All the RasG mutants isolated in different backgrounds grow poorly in shaken suspension, where growth depends on macropinocytosis [10,53,59]. However, in the earliest work, this was ascribed to a defect in cytokinesis. These mutants were unstable [53] and later work with newly isolated RasG− mutants revealed a strong defect in fluid uptake [10]. The fluid uptake defect is the greatest of any single Ras mutant and is not overcome in an enriched medium, indicating that it is mechanistic in nature (figure 3*a,b*). PIP3 patches and macropinosomes form without obvious morphological defect in RasG− cells but at a reduced rate compared to its parent (figure 3*c,d*).

RasS was discovered at the same time as RasC [60]. Macropinocytosis was reduced in the initial knock-out mutants [61], but their speed of migration was increased [62], consistent with the ‘drink or drive’ hypothesis [63] in which macropinocytic cups and pseudopods are seen as competing for the same fixed cytoskeletal resources. A similar, although less extreme, phenotype is found in an RasS− mutant made in the wild-type DdB background [36]. However, when the original laboratory made RasS− cells in a different parental strain, Ax2, the movement phenotypes were completely opposed to those previously reported, though fluid uptake was not measured [64].

We isolated new RasS− mutants in our parental Ax2 and also found no defect in fluid uptake (figure 3*a*,*b*). However, macropinosome formation is reduced in the mutant showing that macropinocytosis is affected. This is presumably compensated for by increased macropinosome size, though this remains to be confirmed. However, when RasS is knocked out in a RasG− background, fluid uptake is further reduced in the double mutant to about a third of the parental rate, as is macropinosome formation. As a control, knocking RasS out in a RasC− background actually increased uptake. Adapting cells to a richer medium does not affect the fluid uptake.

RasB was the third Ras protein to be identified, after RasD and RasG [65]. Unlike the other Ras proteins, which are predominantly at the plasma membrane, a specific antibody localizes RasB to the nucleus during interphase [66]. By contrast, a GFP fusion of RasB localized it to the plasma membrane [28]. RasB interacts with the formin, ForG, which also localizes to macropinosomes and is required for macropinocytosis [28]. These two studies differ in the extent to which axenic growth is impaired in RasB− mutants: workers in Sutherland *et al.* [66] found that growth in liquid medium was severely impaired in a promoter mutant and that null mutants could not be isolated. Workers in Junemann *et al.* [28] could isolate null mutants and found their growth and fluid uptake was only modestly affected (which we have separately confirmed in their mutant, figure 3*a*). However, the uptake of yeast particles was reduced, which correlates with cells forming smaller macropinocytic patches [8,48].

Both studies used variants of Ax2 as parental strain, but whether the differences in growth phenotypes are due to sensitization of one background to loss of RasB, or suppressor mutations in the other is not known. Unfortunately, we have been unable to isolate new RasB− mutants in our standard laboratory Ax2 strain to help resolve the conflict. This work suggests that RasB may have a major role in macropinocytosis, but the conflicting phenotypic data cloud the issue.

It is not known whether activated Rap is recruited to macropinocytic patches or not. Of the three Rap proteins, RapB is unstudied, while a RapC− mutant has no reported defect in axenic growth [67]. RapA is highly expressed in growing cells and thought to be essential. Knockdown by an antisense construct, or overexpression both considerably reduce fluid uptake suggesting RapA is involved in macropinocytosis [68,69].

This work shows that RasG and RasS are important for macropinocytosis, but since a double RasG−/RasS− mutant retains some macropinocytic ability, at least one further Ras protein is likely involved. This may be RasB, or RasD after upregulation in the mutant [56]. RapA also regulates PI3-kinases and its role in macropinocytosis needs exploring further.

## 7. RasGEFs and RasGAPs involved in macropinocytosis

*Dictyostelium* has more than 25 RasGEFs, greatly complicating the task of discerning which are linked to macropinocytosis [70].

RasG is activated by both GefF [71] and GefR [72]. A GefR mutant grows normally in axenic conditions, suggesting no defect in macropinocytosis [70]. GefF together with another RasGEF, GefI, forms part of a complex related to the Sca1 complex that is important in chemotaxis [73]. Our attention was drawn to GefF because attempts to knock it out had failed, suggesting that it might be essential [70] (Thomas D Williams, unpublished observations).

**Figure 4.**
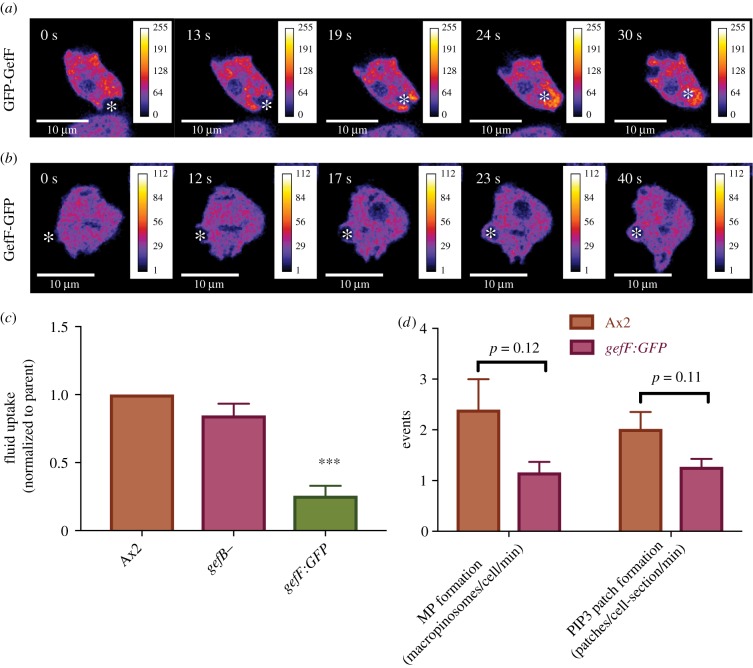
Genetic identification of a RasGEF important for macropinocytosis. (*a*) GFP-GefF localizes to macropinocytic cups in Ax2 cells. The over-expressed reporter is diffusively enriched on macropinocytic cups (asterisk). (*b*) GefF-GFP does not localize to macropinocytic cups suggesting that the fusion protein is not functional. (*c*) Fluid uptake by RasGEF mutants. A GefF mutant created by knocking GFP into the C-terminus of the protein has significantly decreased fluid uptake (pTWGefFknockin); by contrast, there is only a minor decrease in a GefB null mutant. (*d*) The rate of macropinosome and macropinocytic patch formation is decreased in the GefF-GFP mutant.

To gain more information about GefF, we tagged it at either end with GFP. While GFP-GefF broadly and diffusely localizes to the tips of closing macropinosomes, consistent with a role in macropinocytosis (figure 4*a*), the C-terminally tagged protein does not (figure 4*b*), implying that it has a targeting defect. We exploited this possibility by knocking-in GFP at the C-terminus of GefF, thus replacing the endogenous protein with one that cannot localize to macropinosomes. The resulting knock-in mutant is still viable, implying that GefF has retained some function, but fluid uptake is reduced to about one third of wild type [74] (figure 4*c*). This correlates with reduced macropinosome and patch formation (figure 4*c,d*) [74].

GefB is likely the activating GEF for RasS [75] and knocking out GefB in the Ax3 strain, where RasS is the dominant Ras in macropinocytosis, and almost completely abolishes fluid uptake [76]. By contrast, we found no fluid uptake defect when GefB is knocked out in Ax2 (figure 4*c*), similar to the behaviour of RasS− cells in this background. RasB is activated by GefQ, but no defect in macropinocytosis has been reported in GefQ− mutants [77].

The RapGEF, GflB is strongly recruited to macropinocytic cups partially by an N-terminal F-actin-binding domain [78,79]. GflB mutants are severely impaired in macropinocytosis and though macropinocytic cups form, some persist much longer than in the wild type, perhaps because of a defect in their retraction after they have sealed.

Knowledge of the *Dictyostelium* RasGEFs is still fragmentary, but so far as macropinocytosis is concerned it appears that attention should focus on GefF, which activates RasG, GefB, which activates RasS, and GflB, which activates RapA.

Among RasGAPs, NF1 is clearly a central regulator of macropinocytosis as already discussed [48]. Its smaller relative called DdNF1 is involved in chemotaxis, not macropinocytosis [80]. Active Ras is rapidly lost from macropinosomes once they internalize, even in cells lacking NF1, suggesting that at least one more RasGAP must be intimately involved in macropinocytosis, though it's identity is unknown.

## 8. The Rho superfamily

Proteins of the Rho family, including Rac, provide a link to the actin cytoskeleton through their effector proteins. *Dictyostelium* has 20 Rho family proteins (each named Rac) but, with the exception of the Rac subset, they do not fall cleanly into the families recognizable in mammalian cells [81,82]. Comparatively little is known of their individual involvement in macropinocytosis: 13 have been knocked out but only RacC and RacE have growth defects in the liquid medium, which might be linked to cytokinesis rather than macropinocytosis [82,83].

### (a) Rac family

Activated Rac is localized at macropinosomes in *Dictyostelium* [10,23] forming a coincident patch with the active Ras and PIP3 patches. Inhibiting Rac with EHT1864 [84] blocks fluid uptake [8] showing that Rac is required for macropinocytosis, as in mammalian cells [85,86].

Rac1a, Rac1b, Rac1c, RacF1, RacF2 and RacB are all classical Racs, with Rac1a being by far the most highly expressed in cells grown axenically [43] and RacF2 apparently devoted to the sexual cycle [87]. Similar to mammalian cells [88], overexpression of any of the three isoforms of Rac1 (a, b or c), or of activated RacB induces ruffling [89,90]. When activated Rac1 is expressed, numerous arrested macropinocytic cups form but fluid uptake is reduced. This suggests that, though Rac activity is required to form macropinocytic cups, Rac deactivation is required to close them, similar to the situation in some mammalian cells ([86], see also [91]). All of the classical Rac proteins have been knocked out individually with no reported macropinocytosis defect [82,92]; however, multiple mutants to counter redundancy between these similar proteins have not been made.

Activated Rac binds to and activates the PAK protein kinases of which PakC is the most highly expressed in growing cells and binds PIP3 but has only been studied in the context of chemotaxis [93]. PakB is clearly recruited to macropinosomes and while no macropinocytic defect has been reported for the null mutant, expression of an activated form considerably increases macropinocytosis [94]. PakA is recruited to the base of phagocytic cups, but its location in macropinocytic cups is unreported and a null mutant has no defect in fluid uptake [95].

While Rac can be activated downstream of PIP3, by for instance a PIP3-binding GEF, we find that it is still activated within macropinocytic patches of PI3K1-5− cells. These cells form patches of active Ras [10], and a co-incident patch of active Rac is also formed (figure 1*c,d*), although it is possible the relative amount of active Rac is reduced. Thus, it seems likely that as well as activation via PIP3, Rac is also activated from Ras by an independent route.

### (b) Other Rho family proteins

Of the other Rho family proteins studied, RacG is the most similar to Cdc42, which, like Rac1, has been implicated in ruffling for mammalian macropinocytosis [96]. RacG localizes to the plasma membrane, where it accumulates at the rims of large phagosomes, but mutants have no defect in macropinocytosis [97]. A knock-out of RacH has reduced macropinocytosis but, as the protein is localized to intracellular compartments where it is involved in endosome maturation, this phenotype is likely caused indirectly by an endosomal trafficking defect [97]. RacE is also likely not involved in macropinocytosis as the growth defect in null mutants [82] seems to be due to its function in cytokinesis, while fluid uptake is not affected [74,83]. RacC interacts with WASP [98] and overexpression induces ruffling but decreases fluid uptake [69] similar to other Rac proteins involved in macropinocytosis, implying that RacC is involved in bulk fluid uptake.

## 9. Other small G-protein families

The Rab small GTPases are mostly involved in intracellular vesicle trafficking. However, it is worth noting that Rab7 localizes to macropinosomes shortly after internalization and expression of a dominant negative form blocks macropinocytosis most likely because of a vesicle trafficking defect [99]. Expression of dominant negative RabD (Rab14), which promotes lysosome fusion, produces a large reduction in macropinocytosis, presumably also due to a trafficking defect [100,101]. The overexpression of RabS increases macropinocytosis, although the basis for this increase remains unknown [102]. Expression of an activated version of Rab21 causes cells to form more ruffles, giving increased phagocytosis of yeast particles, but no increase in fluid uptake [103].

A knock-out of a Rheb homolog, which is involved in TORC1 activation in mammalian cells, has no defect in macropinocytosis in *Dictyostelium* [104].

## 10. Summary and outlook

Macropinocytic cups in *Dictyostelium* form around signalling patches of PIP3, active-Ras and active-Rac. Patches recruit activators of the Arp2/3 complex to their edges, thus triggering a ring of actin polymerization. Similar arrangements in phagocytic cups, basal actin waves and cell contacts in streams suggest that this ringed actin polymerization may be a general organizing principal of the cytoskeleton enabling cup-shaped structures to be projected from the plasma membrane [10]. While these microscopic observations are very suggestive, evidence of functional importance for the components is also essential.

Ras is strongly implicated in macropinocytosis. Persistent activation, either by removal of the RasGAP NF1 or expression of activated Ras, can strongly stimulate macropinocytosis. The limited genetic analysis of individual Ras proteins also shows that Ras is required for macropinocytosis, with the double-mutant of RasG and RasS having a strong defect in fluid uptake. The cognate RasGEFs remain to be defined but, as well as GefB, GefF is a strong candidate due to the reduced macropinocytosis in a GFP knock-in. NF1 is clearly important, but other RasGAPs must also be involved.

Rac is implicated by inhibitor studies but as yet no single mutant of Rac or the extended Rho family gives a clear macropinocytic defect, so it is likely that multiple members act redundantly.

Much of the genetic information on macropinocytosis in *Dictyostelium* has been gained incidentally in studies focused on chemotaxis. Redundancy and the possibility of modifier and suppressor mutations are further complications. However, recent advances in making multiple mutations and in the manipulation of non-axenic strains should allow a more definitive parts list of the Ras and Rac proteins involved in macropinocytosis and their GEFs and GAPs to be drawn up [36,105].

PIP3 patches appear to be self-organizing [106] and are presumably sustained by positive feedback loops with the aid of the actin cytoskeleton to anchor key components in place, or at least restrict their diffusion [107]. Ras might activate itself through one of its effectors or be activated via PIP3 or Rac; and similarly for PIP3 and Rac. Multiple feedback loops would make the structure robust and may explain why Ras can appear important but not essential in murine embryonic fibroblasts [41], but important and likely essential in *Dictyostelium*.

## Supplementary Material

Supplementary information

## Data accessibility

The datasets supporting this article are available as part of the electronic supplementary material.
