# Supplementary material for: Function of small GTPases in Dictyostelium macropinocytosis
Source: Philos Trans R Soc Lond B Biol Sci. 2018 Dec 17;374(1765):20180150. doi: 10.1098/rstb.2018.0150 (PMC6304742; doi:10.1098/rstb.2018.0150)
Supplement: Supplementary information [file rstb20180150supp1.docx]

**Supplementary Materials**

Function of small GTPases in *Dictyostelium* macropinocytosis

Thomas D. Williams^1^, Peggy I. Paschke^1^, Robert R. Kay^1^

*^1^MRC-Laboratory of Molecular Biology, Francis Crick Avenue, Cambridge, CB2 0QH, UK.*

**Table 1: *Dictyostelium* strains used in this work**

| **Strain** | **Parent strain** | **Genotype** | **Source** |
| --- | --- | --- | --- |
| Ax2 (Ka) | DdB |  | Kay Lab |
| DdB | NC4 |  | Dennis Welker |
| HM1895 | Ax2-214 | *rasB-* | [1] |
| HM1800 | Ax2 (Ka) | *rasC-* | this work |
| HM1726 | Ax2 (Ka) | *rasG- flox* | [2] |
| HM1867 | Ax2 (Ka) | *rasS-* | this work |
| HM1981 | Ax2 (Ka) | *rasC-,rasS-* | this work |
| HM1855 | Ax2 (Ka) | *rasG-,rasS-* | this work |
| HM1784 | Ax2 (Ka) | *gefB-* | this work |
| HM1883 | Ax2 (Ka) | *gefF:GFP (KI)* | [3] |
| HM1200 | Ax2 (Ka) | *pikA-, pikB-, pikC-, pikF,*  *pikF-,pikG- (pi3K1-5-)* | [4] |

**Table 2: Reporter plasmids used in this work**

| **Plasmid** | **Marker 1** | **Marker 2** | **Selection** | **Source** |
| --- | --- | --- | --- | --- |
| pPI32 | GFP-CorA | PH-Akt-mCherry | G418 | this work |
| pTW007 | PH-PkgE-mCherry | N/A | G418 | this work |
| pTW007Hyg | PH-PkgE-mCherry | N/A | Hygromycin | this work |
| pTW030 | GFP-GefF | N/A | G418 | this work |
| pTW031 | GefF-GFP | N/A | G418 | this work |
| pPI432 | GFP-RasG | N/A | G418 | this work |
| pPI332 | GFP-RasG G12T | N/A | G418 | this work |
| pPI434 | GFP-RasS | N/A | G418 | this work |
| pPI445 | GFP-RasS G12V | N/A | G418 | this work |
| pPI363 | GFP-RasG G12T | PH-PkgE-mCherry | G418 | this work |
| pTW050 | GFP-RasG G12T | PakB-CRIB-mCherry | G418 | this work |
| pDM1140 | Raf1-RBD-mCherry | PakB-CRIB-GFP | G418 | [2] |

**Table 3: Details of knock-out and knock-in constructs**

The target gene, construct name, selectable marker, source and the sequences used to amplify the 5’ and 3’ homology arms (from which the deletion can be deduced) of the construct are given.

| **Gene** | **Vector** | **Selection** | **Source** | **5’ arm** | **3’ arm** |
| --- | --- | --- | --- | --- | --- |
| ***gefB*** | pGefBKO | Blasticidin | this work | 1.ACCCGGGTGGTATCCAGGAATTTACATAGGAA  2.GATAGCTCTGCCTACTGAAGTGGTTAGAAGAAGAAAAGGAAGACA | 1.CTACTGGAGTATCCAAGCTGATTAGATTTTCAACACACAAGCACA  2.ACCCGGGGTGAGAGTGAAGTGAGTGAAAGTGA |
| ***gefF (KI)*** | pGefFGFPKI | G418 | this work | 1.GGATCCAGGAAATTCTATATTATTTTGGGGTGG  2.ACTAGTTAATTTAACAATTGGACTTAAACTATTTG | 1.CTCGAGGACTCATCTCTTTTTTTAAATGTATC  2.GAGCTCAGTGAAACGGATATCATTGAAACTATTAATG |
| ***rasC*** | pRasCKO | Blasticidin | Evgeny Zatulovskiy | 1.AAGGGGCCCGTAAGGGTGCAGTATTTACAATTCATGGTGC  2.TTGAAGCTTAAGTGCCGATTTACCAACACCACCATCACCAACG | 1.GCGGCCGCCTTTACCCTCGTTAGAGAAATTAAAAGATGG  2.CCGCGGTTATTTGTGCCAATTTGTTAGAAAGTGTTTCC |
| ***rasG*** | pRRK1 |  | [2] | 1.TTAACTGAAAAAAACAGCGTAGTATGAAATCGG  2.CTTGGATTAATTGAATGGTTAAGGCACTTTTACCGAC | 1.GTTGAGGAAGCTTTCTATTCACTCGTACGTG  2.GGTAGTGATAATATTTTTCAACACTTTCAATGAG |
| ***rasS*** | pRasSKO | Hygromycin | [5] | 1.GCCAGCTGCTAGATATAACCGACTCTATC  2.GATAGCTCTGCCTACTGAAGGATTTACCAACACCACCTGGTCCAAC | 1.CTACTGGAGTATCCAAGCTGCTCAAGAACAAAATACTGATCAAC  2.GGCAGCTGCATGGTGAATTCAAGATGAACATG |

**Table 4: Primers used to screen for gene modifications**

Three different types of PCR product were used to screen for homologous events and for removal of the selection cassette following expression of Cre recombinase. ‘Casette-flank’ employed one primer from within the selection cassette and the other from the flanking regions beyond the extent of the vector arms; ‘Span’ employed 5’ and 3’ primers beyond the vector arms and ‘Internal’ had both primers from within the vector and across the deletion.

| **Gene** | **Product type** | **Primer sequence** |
| --- | --- | --- |
|  | Cassette-flank | 1.CGAATACTTCTATCTACTTCGTCAGAATA  2.GTTAAAGATAAATTTCAAGATGTTCGTAA |
| ***rasC*** | Span | 1. TGGTAAATCGGCACTTACTATTCAAT  2. GTGGTACTTTTCTATCTTTGTCTGGT |
|  | Internal | 1.CTCTTATCGTAAACAAGTAAACATTGATGAAGAAG  2. GATCTAATATATTGATCTCTCATAGCGCTATACTC |
|  | Cassette-flank | N/A |
| ***rasG*** | Span | N/A |
|  | Internal | 1.CCGCATTTTTTGGCATTCGCAACACCC  2.GAGTACAAGCTTTTAATGGTCTCTTCTTC |
|  | Cassette-flank | 1.CTACTGGAGTATCCAAGCTG  2.CAAGAGGCAAGAGAATTTGTATCATTCATG |
| ***rasS*** | Span | 1.CATATTGAAATCTAGATATAACCGAC  2.CAAGAGGCAAGAGAATTTGTATCATTCATG |
|  | Internal | N/A |
|  | Cassette-flank | 1.CGAATACTTCTATCTACTTCGTCAGAATA  2.ATTAGTATCAGCTACTCTAACACAAGATG |
| ***gefB*** | Span | N/A |
|  | Internal | 1. GATTTGGTGTTGATGAATTTGGTG  2. CCTGCTTTGAAAATGATGAAATCTCC |
|  | Cassette-flank | 1.CTACTGGAGTATCCAAGCTG  2.TTCAATTAAAGAAGCACCAGAGATTG |
| ***gefF (kI)*** | Span | 1.TTGTCCAAAATCGTATTTCACATTC  2. TTCAATTAAAGAAGCACCAGAGATTG |
|  | Internal | N/A |

**Figure S1: Examples of diagnostic PCRs**

PCRs were conducted on DNA from parental Ax2 and mutant strains using the pairs of primers indicated in Table 4.

**RasC**


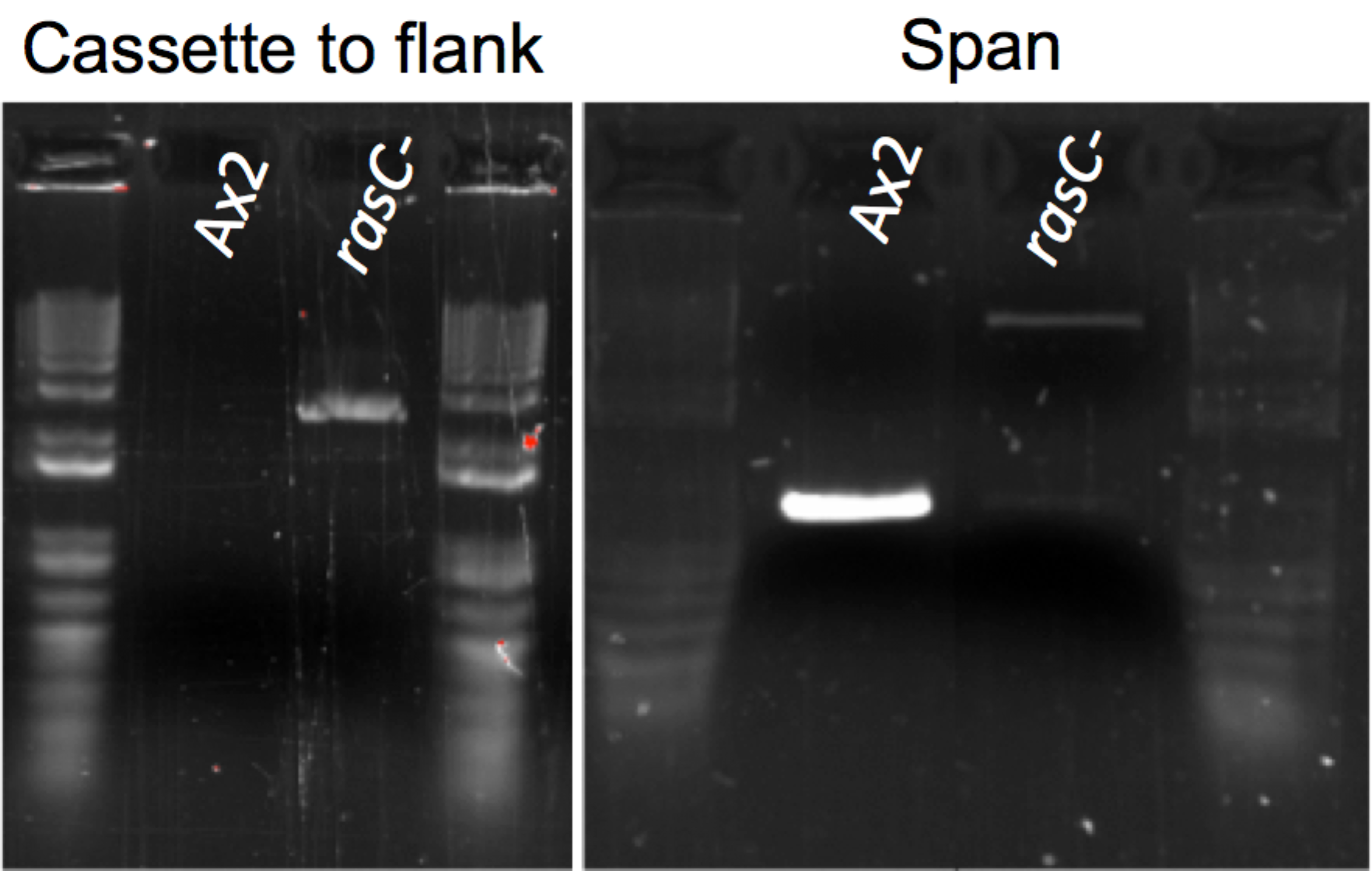


**RasG**


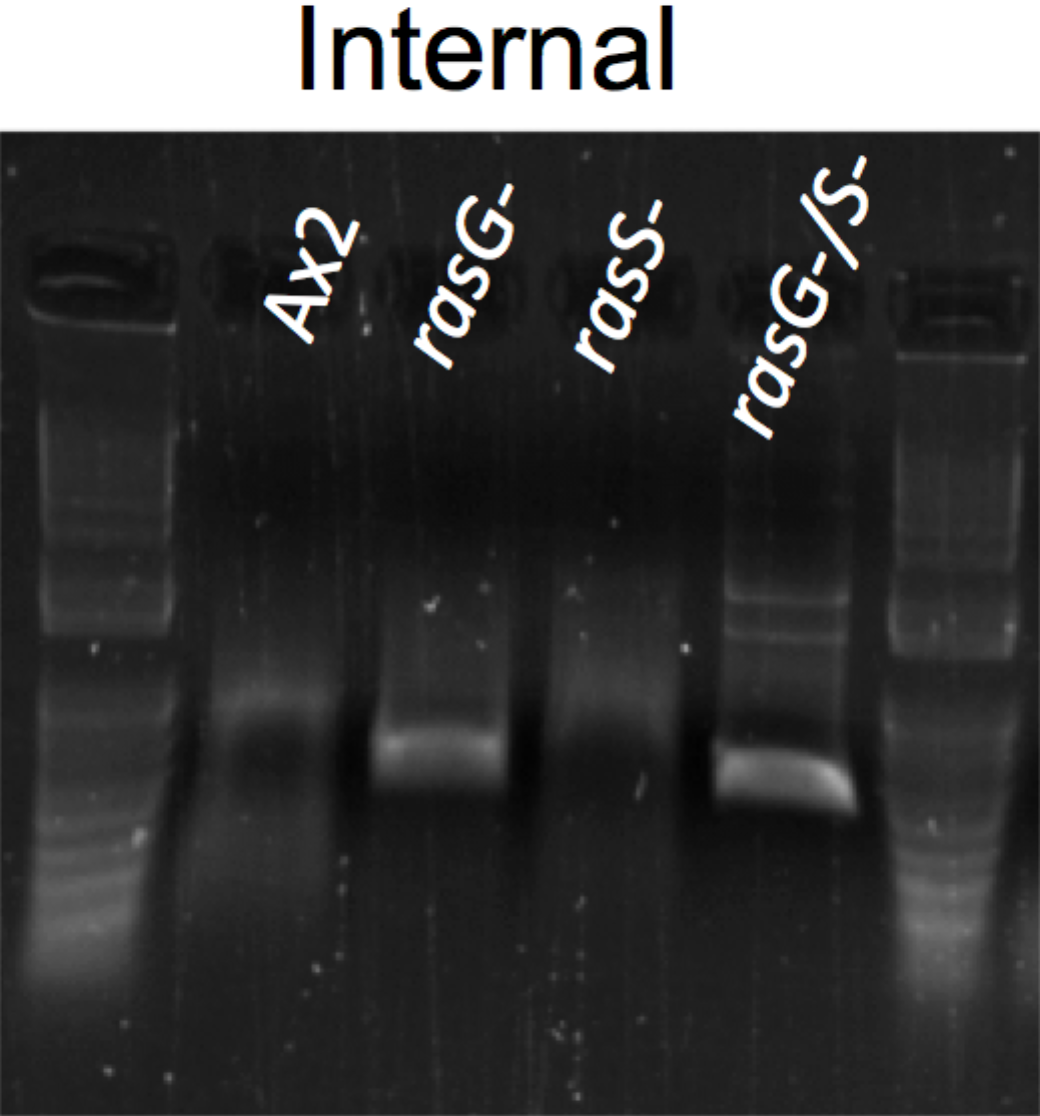


**RasS**

**
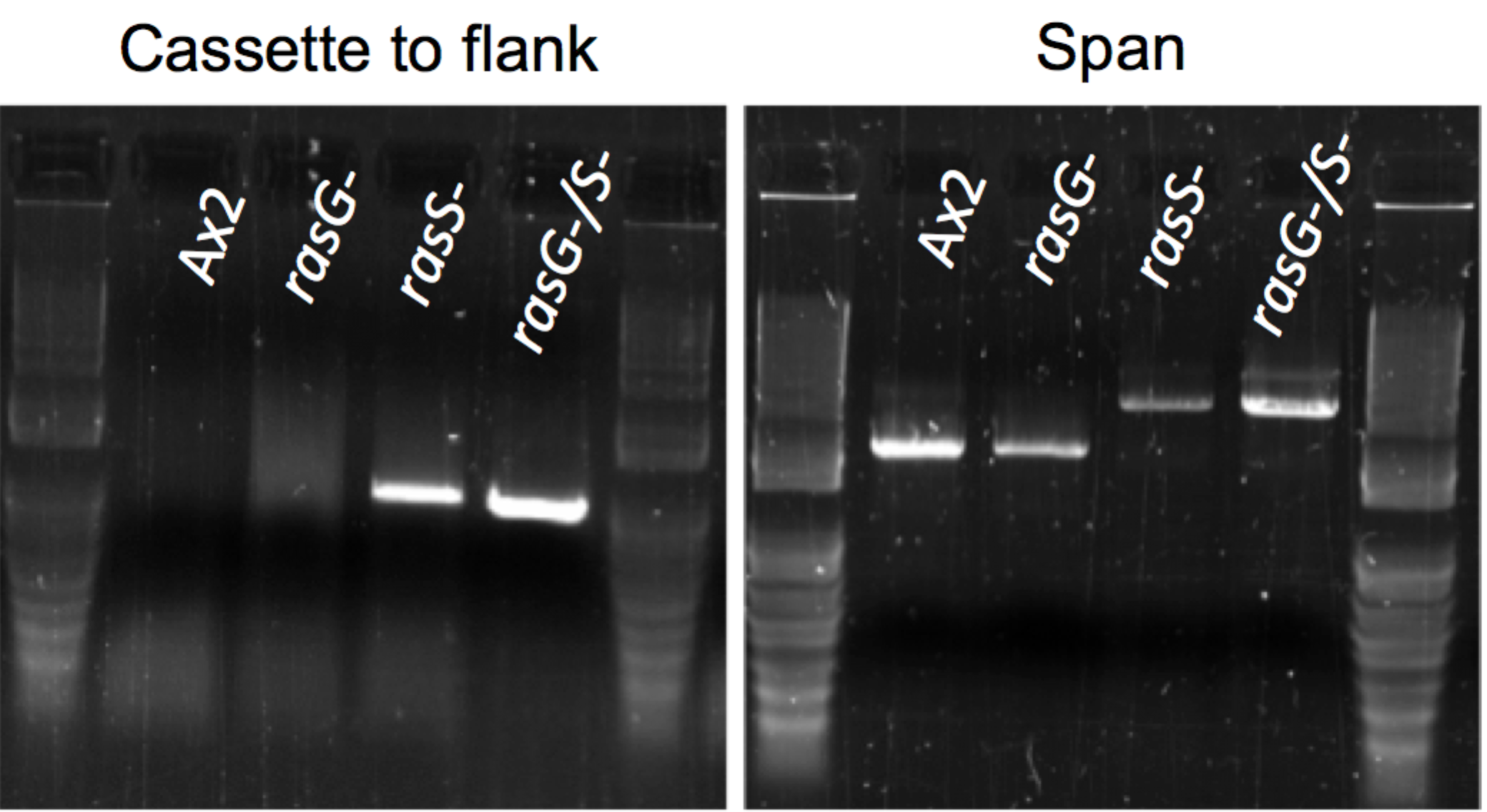
**

**GefB**

**
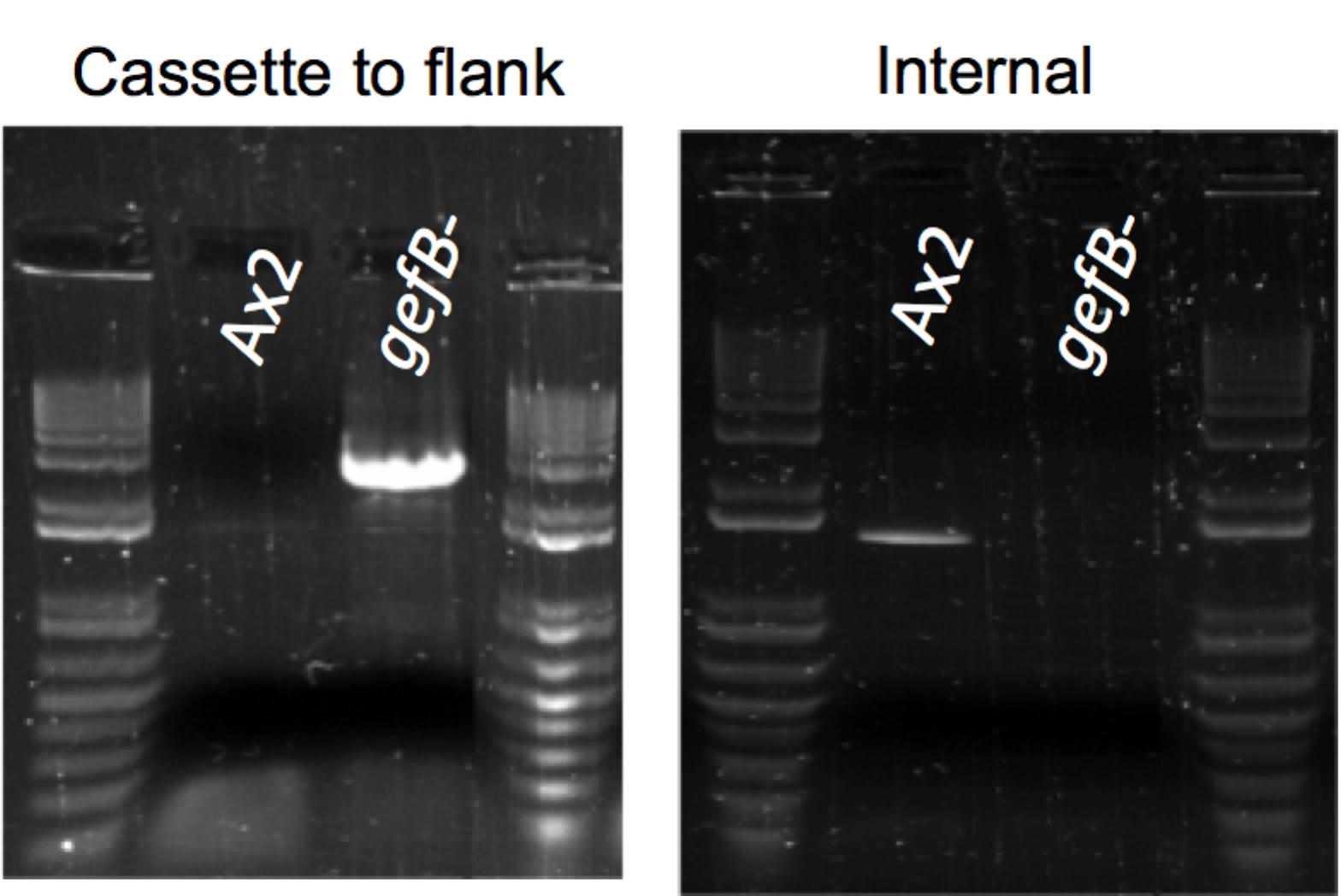
**

**GefF:GFP knock-in**

**
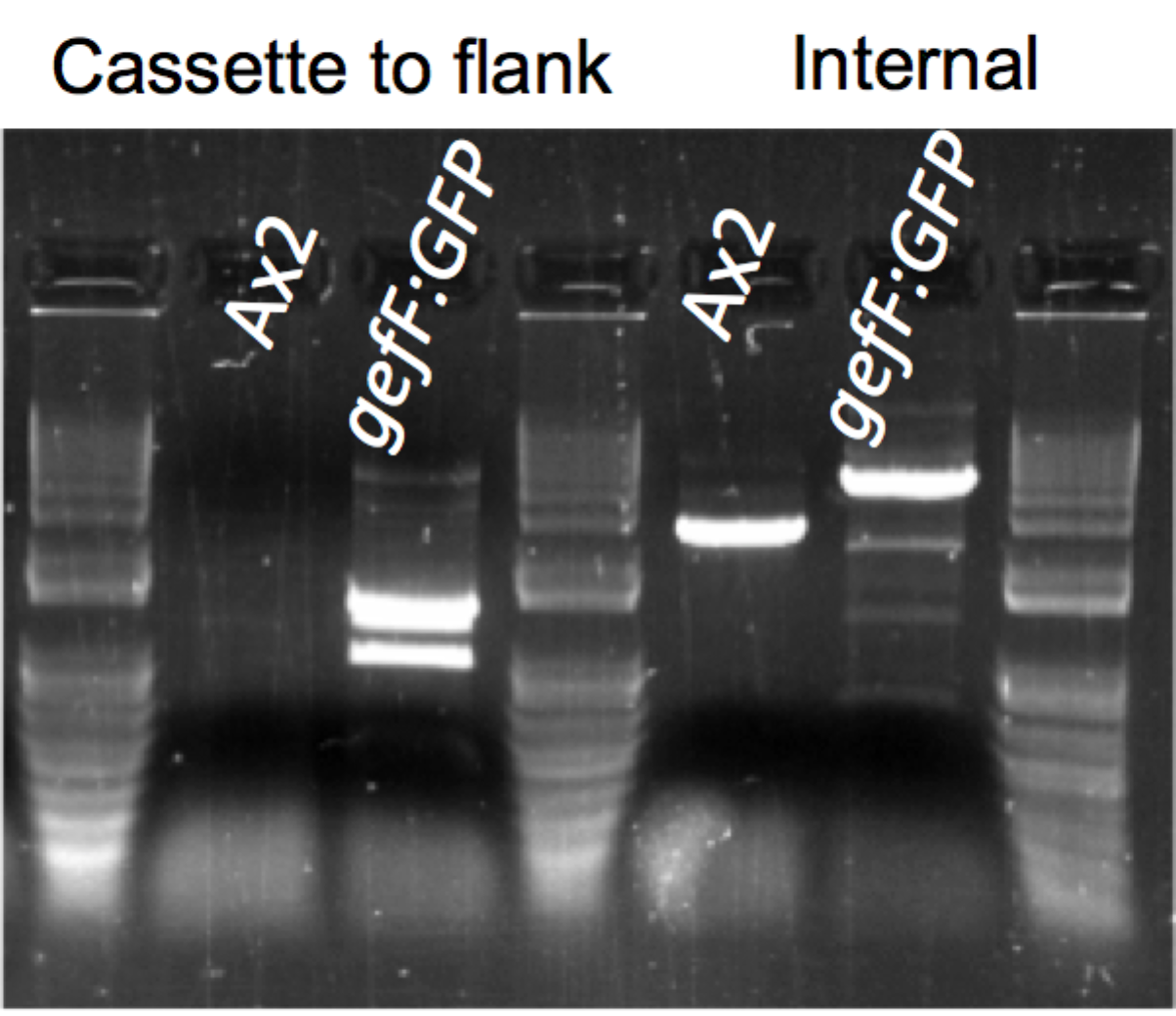
**

**References for Supplementary materials**

1. Junemann, A., Filic, V., Winterhoff, M., Nordholz, B., Litschko, C., Schwellenbach, H., Stephan, T., Weber, I., and Faix, J. (2016). A Diaphanous-related formin links Ras signaling directly to actin assembly in macropinocytosis and phagocytosis. Proc Natl Acad Sci U S A *113*, E7464-E7473.

2. Veltman, D.M., Williams, T.D., Bloomfield, G., Chen, B.C., Betzig, E., Insall, R.H., and Kay, R.R. (2016). A plasma membrane template for macropinocytic cups. eLife *5*.

3. Williams, T.D. (2017). A molecular genetic investigation of *Dictyostelium* macropinocytosis. PhD Thesis. In MRC Laboratory of Molecular Biology. (Cambridge: University of Cambridge).

4. Hoeller, O., and Kay, R.R. (2007). Chemotaxis in the absence of PIP3 gradients. Curr. Biol. *17*, 813-817.

5. Paschke, P., Knecht, D.A., Silale, A., Traynor, D., Williams, T.D., Thomason, P.A., Insall, R.H., Chubb, J.R., Kay, R.R., and Veltman, D.M. (2018). Rapid and efficient genetic engineering of both wild type and axenic strains of *Dictyostelium discoideum*. PLoS One *13*, e0196809.
